# Social Determinants of Discrimination and Access to Health Care Among Transgender Women in Oregon

**DOI:** 10.1089/trgh.2019.0090

**Published:** 2020-12-11

**Authors:** Jonathan Garcia, Richard A. Crosby

**Affiliations:** ^1^ College of Public Health and Human Sciences, Corvallis, Oregon, USA.; ^2^ College of Public Health, University of Kentucky, Lexington, Kentucky, USA.

**Keywords:** discrimination, gender-affirming health, Oregon, social determinants, transgender women

## Abstract

**Purpose:** Transgender women in the United States experience health disparities and limited access to gender-affirming health services. This study describes the social determinants of health that shape access to health services for transgender women in Oregon, a state with a high tally of gender-affirming policies.

**Methods:** We conducted qualitative interviews with 25 transgender women between 18 and 39 years of age. Interviews explored the social, economic, cultural, and legal factors that shape access to health. A Qualtrics survey captured sociodemographic characteristics. We identified facilitators and barriers to accessing gender-affirming services using thematic analysis of qualitative data.

**Results:** Our participants perceived gender-affirming health services in Oregon to be relatively trans-friendly, compared to other parts of the United States. This perception drew several transgender women in our sample to migrate to Oregon from other “more conservative” states. Facilitators included ease with legal name change (60% had completed), inclusiveness of hormone therapy in the Oregon Health Plan, and availability of informed consent hormone therapy. However, for our participants, economic and social discrimination were major limiting factors to accessing and navigating health services. Social factors exacerbated difficulties navigating and understanding health systems to achieve coverage; 20% had insurance that did not cover hormone therapy. Specialized surgeons were located in urban/suburban centers; electrolysis coverage was limited; and 10% had gender-affirming surgery.

**Conclusion:** This study indicates that services are necessary to assist with navigating access to gender-affirming health care, even in affirming policy contexts like Oregon.

## Introduction

In the United States, despite legal protections for the lesbian, gay, bisexual, transgender and queer (LGBTQ) population, sparse legislation protects transgender women from discrimination, including employment and housing discrimination.^1,2^ Consequently, transgender women are prone to live in poverty and experience unemployment and homelessness; and they may have issues accessing transgender-related health care.^3–6^ Furthermore, it is likely that these experiences culminate in chronic daily stress and in a host of negative mental and physical health outcomes.^7–10^ Despite adverse health outcomes, transgender women may experience excessive barriers to obtaining health care and transgender-related services, including mental health services.^11–15^ Sevelius et al.^15^ concluded that gender-affirming health care may be the most critical of all factors. Gender affirmation is described as validating one's gender identity and expression, and this typically involves having a body image that is concordant with gender identity, as well as social recognition and legitimacy and acceptance of the self.^16–19^

According to the social determinants of health (SDH) framework of the World Health Organization, health outcomes are determined by

the conditions in which people are born, grow, work, live, and age, and the wider set of forces and systems shaping the conditions of daily life. These forces and systems include economic policies and systems, development agendas, social norms, social policies, and political systems.^20^

Gender inequity is a key social determinant. Although most applications of the SDH have referred to inequities that cisgender women experience, a budding literature has started to apply the SDH to transgender identity and health.^16,21–23^ Martinez-Velez et al. indicate that “the further an individual's gender expression shifts away from the stereotypical gender binary constructs, the higher degree of violence, social rejection, and hostility they may face” and that these “experiences are enacted through family rejection, social marginalization, and institutional discrimination at all levels”.^22, p. 10^ For transgender women, the SDH are thus shaped by both gender oppression and by social rejection of their gender transgression. Studies have focused on the need for structural interventions to grant transgender and gender nonconforming population access to health services and for gender affirmation in legal documents.^16,22^ However, research guided by critical theory highlights that reducing womanhood to documentation may reinforce transmisogyny^24^ and that legal changes that occur without changes in social norms mask underlying injustices that persist in daily life.^25^ For transgender women, “passing” and “visual conformity” to feminine gender norms can determine housing and employment opportunities.^26–28^ One of the SDH's major contributions to health promotion is its attention to the structural and policy factors that determine proximal health outcomes. However, less consideration has been given to the factors, such as social, economic, and geographic marginalization, that facilitate and impede the implementation of new gender-affirming policies and systems in the daily lives of transgender people.

This study addresses this gap in the literature on the SDH by examining the experience of transgender women in Oregon, which is among the states with the most gender-affirming policies. According to a recent study conducted by the Movement Advancement Project, Oregon is ranked as one of the 14 states with high overall LGBTQ Policy Tally and high Gender Identity Policy Tally in the United States, which are “indicative of significant progress toward LGBTQ equality across many policy areas, typically including nondiscrimination, LGBTQ youth, health care, criminal justice”.^29, p. 1^ As of January 1, 2015, the Oregon Health Plan (OHP; Oregon's version of Medicaid) is required to pay for medically necessary transgender health care services.^30^ In 2017, Oregon policies relative to name changes and gender changes on legal documents allowed residents to identify as nonbinary, neither male nor female, on their driver licenses and identification cards.^31^ By studying challenges to accessing gender-affirming care in an affirming policy context, we may begin to reveal social and cultural determinants of health that influence policy implementation. Accordingly, the purpose of this study was to gain an in-depth understanding of social determinants of access to gender-affirming health services among transgender women residing in a state with relatively affirming policies.

## Methods

### Study sample and recruitment

Individual in-depth interviews were completed with 25 transgender women between November 2017 and April of 2018. Recruitment occurred over the span of 6 months (active recruitment and interviewing) in three Oregon cities: Corvallis, Eugene, and Portland. These cities were selected in consultation with trans community leaders. Portland (largest population in Oregon: 648,740) and Eugene (second largest population: 169,695) have major medical centers with trans-related services; Corvallis, a large town (10th largest population: 59,280), is known to have a progressive social environment.^32–34^ In one recruitment method, we consulted with leaders of the trans community and local health care agencies. They referred transgender women to one of the two researchers (coauthors). Leaders were identified through community organizations (e.g., Pride Center, community health clinics, online social groups, word-of-mouth). A second method involved posting the study flyer in Facebook groups to which trans community leaders belonged. The flyer included a description of the inclusion criteria, and participants were asked to self-identify as potential participants. Inclusion criteria were as follows: self-identification as a transgender woman, being between 18 and 39 years of age, and currently residing in Oregon. We selected adult transgender women in this age range based on previous research conducted by the authors indicating their exposure to social and structural barriers to accessing gender-affirming care.^35,36^ Twenty-five eligible volunteers provided verbal informed consent and completed the interview process, which occurred in a semiprivate but public area of their own selection. Participants were compensated for their time, emotional and intellectual labor, and transportation with a $50 gift card.

### Assessment

All assessments were conducted on a one-to-one basis. First, a brief tablet-based, self-administered questionnaire was used to collect basic sociodemographic information. Then, one of the two researchers engaged the volunteer in a semistructured, qualitative interview designed to elicit experiences with discrimination, social support, resilience, and gender-affirming health care. Interviews assessed perceptions of the social environment and how it influenced mental health and physical health. Similarly, we assessed women's perceptions of the legal, policy, and economic environments. Interviewers recorded interviews in the form of verbatim notes. This strategy improved our ability to establish trust with participants and allowed us to conduct interviews in public spaces of their choice. The social identities of the coauthors facilitated rapport and knowledge of the community; one author is a cisgender, gay, and Latinx man, and the second is nonbinary and White.

### Data analysis

Data were analyzed using immersion crystallization,^37,38^ which is “a dual process involving detailed review of textual data and momentarily suspending the immersion process to reflect on emerging findings until consistent themes are identified”.^39^ First, we used within-case analysis. We produced detailed reports of each interview we conducted, coded quotes from the interviewees' narratives into major *a priori* thematic categories (e.g., social support, discrimination, resilience, and gender-affirming health care). We discussed cases during the process of data collection, allowing us to begin to see emerging themes (e.g., queer migration). The iterative process of data collection and analysis allowed us to determine when data saturation had been achieved. At this point, we were no longer observing new patterns or thematic nuances when conducting additional interviews. The next phase of data analysis occurred when we had concluded data collection. In this phase, we exported sociodemographic data from the Qualtrics questionnaire into Excel and conducted basic analyses of frequencies and averages to characterize the sample. We explored patterns within each theme across participants. This required reorganizing data into thematic documents that brought together quotes and overarching analytic memos. Study procedures were reviewed and approved by the Institutional Review Board at Oregon State University.

## Results

The mean age of our sample was 27.56 years. The mean age when they came out to anyone about their gender identity was 22.16 years. Most participants identified as White (21 out of 25), 3 identified as Asian, and 1 identified as a Native American. Nearly a quarter of the sample (*n*=6) was homeless in the past 12 months, 76% had completed at least some college education, and only 20% had full time employment at the time of the study. All of our study participants had health insurance, which was acquired through OHP (Medicaid), parents' insurance, school insurance policies, or through other private insurance. Our sample includes a diverse range of sexual orientations (Table 1).

**Table 1.** Sample Characteristics

Age (mean, SD)	27.56 Years (6.21 Years)
Education % (*n*)	4% (1) Some high school
	20% (5) Graduated high school or GED
	40% (10) Some college, assoc. or tech. degree
	16% (4) Bachelor's degree
	20% (5) Any post graduate studies
Employment % (*n*)	20% (5) Full-time
	28% (7) Part-time
	16% (4) Unemployed
	8% (2) Homemaker
	8% (2) FT student
	8% (2) Disabled
	12% (3) Other
Sexual orientation % (n)	4% (1) Heterosexual woman
(“Other/Multiple” included: Homoflexible lesbian, Pansexual Trans Femme, Femme Agender, Panromantic Asexual, Nonbinary, Woman-Loving-Women, Queer Woman, “Just Me”)	32% (8) Lesbian woman
	20% (5) Bisexual woman
	20% (5) Pansexual woman
	24% (6) Other/Multiple

FT, full time; GED, general education development certification; SD, standard deviation.

### Social support coupled with ongoing discrimination

Although Oregon has a gender-affirming policy context, participants reported relying on informational, material, and emotional support from transgender friends to cope with ongoing social discrimination. A 21-year-old pansexual woman explained,

I'm happy spending time with friends and family, self-actualizing, working toward my own goals, a lot of art, drawing, painting, sculpture; working toward aligning my lifestyle with my ideals. This includes my interactions with others and activism. I'm part of the Democratic Socialists. Having a good safety net helps. Family and financial support has helped me develop good self-worth. I'm not internalizing other peoples' prejudice of me.

Several quotes (Q1–Q5) presented in Table 2 are illustrative of how social support helped participants cope with discrimination. A 21-year-old woman-loving-woman describes her trans friends as a major source of support, especially when she experiences “transphobic things…like misgendering in the streets” and “restaurants”; she emphasized “otherwise, it's death by a thousand cuts, wearing you down and invalidating you.” Participants who belonged to networks of trans people were able to navigate transphobia, and some, like this 28-year-old gender-fluid lesbian woman saw their contribution to the well-being of young transgender women as a source of strength. She explained,

**Table 2.** Experience of Social Support and Discrimination for Transgender Women in Oregon

Theme	Representative Quotes
Social support and resilience	Q1 My friends act as a bubble around me and make it easier. (21 years old, bisexual woman)
	Q2 Friends enrich my life. We ground each other, so we know things are going to be OK. Most of my friends are trans. We talk about transphobic things that happen to us, like misgendering in the streets, at restaurants. Your suffering is valid but it isn't forever. Otherwise it's death by a thousand cuts, wearing you down and invalidating you. (21 years old, woman-loving-woman)
	Q3 I grew up in Albany, Oregon, like one of 20 Asian kids there, and felt a lot of tokenization. I started meeting other trans women who told me, “hey you don't have to fit into roles.” Then, I started wearing skirts, and met my current roommate and best friend through another friend in the [trans punk] music scene. (22 years old, lesbian woman)
	Q4 The communities that I am in make me happy. I am in BDSM and kink communities, I am also poly-amorous. I discovered my trans-ness through these communities. I am also a painter and I do drag art –my friends and family support me and care about me. I have really great support from my chosen family. I have a strong poly community that gives me tremendous support. Being trans helped me learn to reach out to the community for help and support. (27 years old, Pansexual Trans Femme)
	Q5 I like to be with people who bring me peace and happiness – I have come to enjoy life based on this. I depend on my faith – being trans actually helped me find direction in life. This is especially the case relative to my relationship with other trans-people. In essence, being marginalized has actually enriched my life. I always like to think that future trans-kids have it easier based on my work in the trans communities. People I know help me stay strong. I do not always deal well with adversity. My faith helps me. My emotions can be a mixed bag – I often just hide under a blanket and escape from the world. (28 years old, gender-fluid/transgender lesbian woman)
Employment discrimination	Q6 A week after I came out as trans I was let go. The manager told me, “your anger problem creates a hostile work environment.” It was a typical male environment. I might have even told a few transphobic jokes myself before coming out. (26 years old, lesbian woman)
	Q7 I have had problems getting a job. I do housekeeping and cleaning but employers keep asking during interviews if this is how I will look at work… For example, at my last job they tried to fire me because of being a trans woman. They branded me as a trouble maker, I was sexually harassed, etc. I reported this to the Human Resources office – they in turn laid 50% of the blame on my “choice” to be a trans woman. They kept assigned my work that required me to change clothes at-work – knowing this would be a problem for me. I was fired and have been in a lawsuit now with this for 1.5 years. The employer seems to quickly accept trans men, they only have an issue with trans women. I confront men who deny my gender – this may happen in places like a store where I am shopping and people in line are called sir, miss, mamm, etc., but my gender is not acknowledged at all. I will turn these people in to the store manager. These and other micro-aggressions take a toll on me. I have been refused service at a pizza place based on being a trans woman. (30 years old, bisexual woman)
	Q8 I have not worked in 3.5 years. I had issues at job interviews due to being trans. I gave up trying 2 years ago. I have never been fired from a job. I have been evicted from a residence. This was based on being accused by neighbors of being a trans-hooker. I was called a street walker. The property manager evicted me based on being the protagonist in all of this. The real issue was my clothing. (32 years old, Femme Agender/panromantic asexual).
“Coded” and direct social discrimination	Q9 In Oregon I've experienced more coded discrimination… where a person doesn't necessarily know that what they are doing is discrimination, but to them I seem foreign, scary and different. A lot of people don't treat me as a true woman because I don't pass as well. (19 years old, bisexual woman)
	Q10 I was socially discriminated against in high-school. I was out but had very little support. I was (and continued to be) in fights with doctors over my transition needs – the burden of proof was also on me – getting hormone blockers was a struggle. My identification now has the correct gender, but this was huge issue before. I had a co-worker who I really liked and respected, she was surprised when I came out. Now, I do experience privileges that come with being a heterosexual woman – I am one of many lesbian wives and I hang with other lesbian wives. I like being a feminist academic. There are few social options for me in Eugene – it is not the queer mecca that people think. (29 years old, bisexual woman)
	Q11 I was at dead end jobs where I was dead-named and sired. I work as a residential caregiver. The staff, mostly cismen, asked if I got [bottom] surgery. At least 5 men have asked if I got surgery. I felt very uncomfortable, really shitty, ruins my whole mood. Why did you remind me? Really, you want to talk about my genitals? (22 years old, lesbian woman)
	Q12 I live in a gender equity floor of my college dorm. It's overwhelmingly transmasculine. Trans women are the minority and are forgotten or overlooked even in these spaces. Trans guys take about oppression and they ignore me or interrupt me, saying “trans women have male privilege,” which below the surface reminds you that you don't belong. The trans resources here are skewed toward trans guys. (21 years old, woman-loving-woman)
	Q13 Eugene is a polarized place when it comes to trans-discrimination. About 50% of the people are trans-friendly and the other 50% are transphobic. In an apartment complex where I lived once people were putting Nazi symbols around my place and leaving urine and other weird stuff on my door. This was a type of hate crime. I had a knife pulled on me and they threatened my dog. The superintendent was yelling at me and blaming me for this. (30 years old, lesbian woman)
Police discrimination and mistrust	Q14 Eugene is not as trans-friendly as everyone thinks. It is hard to know who is genuine and accepting. I am very careful not to take chances – too many people are transphobic… I have been a victim of police harassment. They misgender on purpose in attempt to try to bait me into a reprisal. They antagonize me intentionally. Many people know my dead name and they use it (including police) to insult me. (32 years old, Femme Agender/panromantic asexual).
	Q15 I first came out to my gay cousin – he then threatened me with a gun. I was locked in a room (damp, with shit and urine) for 3 days – this happened in a rural area. I could not tell the police because they are too corrupt to trust. (37 years old, pansexual woman)

BDSM, bondage, discipline, domination, submission, sadism and masochism.

Being trans actually helped me find direction in life. This is especially the case relative to my relationship with other trans people. In essence, being marginalized has actually enriched my life. I always like to think that future trans-kids have it easier based on my work in the trans communities. People I know help me stay strong. I do not always deal well with adversity. My faith helps me. My emotions can be a mixed bag – I often just hide under a blanket and escape from the world.

Moreover, several women in our sample (*n*=8) were drawn to Oregon because of its reputation as a welcoming and “trans-friendly” state. A 19-year-old bisexual woman claimed that this is the reason she moved to Oregon from Wyoming.

While Oregon has some problems, it's incredibly good compared to Wyoming. The health care system here is so much more open [for transgender women] and available; I've faced less discrimination. I chose Oregon because it's really solid for transgender people to live, especially with informed consent clinics for hormones where you don't need a note from your psychologist. I'm on my mom's insurance (from Wyoming), which doesn't cover anything trans-related; and I don't have OHP yet because I'm still not considered a resident.

Another participant who moved to Oregon expressed a similar view.

Getting out … was the key for me. I have not been victimized in Oregon. I did get beat up a lot because of being a trans woman in high school there… This was all because of being a trans woman. I had self-destructive tendencies, but the hospital there would not take trans patients. I have had few opportunities as a trans woman and trans-friendly environments have been rare for me. (32 years old, lesbian woman)

This subsample of participants believed that they experienced fewer instances of discrimination in Oregon relative to other states, which included Wyoming, Montana, Mississippi, and Idaho (see Q34 and Q35 in Table 3).

**Table 3.** Facilitators and Barriers to Access Trans-Specific Health Services

Theme	Representative Quotes
Specialist physicians	Q16 I had to figure out the steps. First, I found a good gender therapist, but I had to pay out of pocket because it wasn't covered in my town. I had 5 sessions but therapist couldn't diagnose me with gender dysphoria, so I had to see a psychiatrist in Eugene to get a letter with the diagnosis. Then I had to see an endocrinologist in a women's health care clinic and a primary care physician who misnamed me. (21 years old, bisexual woman)
	Q17 Now it's harder that I'm on my own to find doctors, and it's really hard for my friends who are not on their parent's insurance or on OHP, or who can't find doctors willing to prescribe the right medications or procedures. For genital reconstruction there is a two-year wait, and I have a friend who is extremely gender dysphoric about that. On top of mental health difficulties and living expenses it's hard to take care of all that stuff at once. (22 years old, lesbian woman)
	Q18 There were no doctors in Grand Ronde so I had to travel 1 hour and 45 minutes by car or 3 hours by public transit to get to my gynecologist. (22 years old, pansexual transgender woman)
	Q19 For surgery it is pretty difficult. It takes several years on the waitlist, and it's like only one doctor takes OHP. (24 years old, lesbian/nonbinary)
	Q20 My health care in Eugene was OK – it is easy to be on Medicaid in OR. Two places in Eugene offer trans health care – one is understaffed and provides middle of the road care – the other is about the same. I keep having to educate the doctors about trans women health care and transition care – bottom surgeries here are a problem. I deal with adversity by an activist in regards to health care for trans women. I also deal with adversity by leaving places that do not provide my health care needs. If I focus on getting mad that activism helps me cope. I am becoming burned out on this, however. I have had to obtain my trans-related meds on my own, when I lived in small town in another rural state. I managed my own transition until I moved to Seattle where I saw my first-ever endocrinologist. (32 years old, lesbian woman)
HRT	Q21 I'm on HRT but don't have a doctor in the area. There are a lot of hoops and gatekeeping. (22 years old, lesbian woman)
	Q22 I was able to get on hormones easily because I sought out someone who does informed consent instead of WPATH. Every trans person I know hates it [WPATH] because they [doctors] think they can determine our own gender better than we can. (24 years old, lesbian/nonbinary)
	Q23 My challenges have been informed consent re: transitioning care – doctors who come across as knowledgeable are often not at all in the know. For instance, my current doctor doubled the amount of my hormone cream recently – I had to cut it back by half. I had bad mood swings – it made emotional stability very difficult. Doctors should know what they are doing—but they pretend to know. (29 years old, bisexual woman)
Electrolysis	Q24 I was going to get a vaginoplasty but it requires having electrolysis. I keep getting the run around. My case manager is helping but prior authorization is not coming through; they have requested it twice. One electrolysis specialist that was in network is retiring months before I can see her. If I can't get electrolysis done by when they scheduled the surgery, it will get pushed back. (26 years old, lesbian woman)
	Q25 Having to educate insurance people was frustrating. I had to learn insurance codes and a lot of jargon that insurance uses to file appeals for electrolysis and then the place closed. (23 years old, heterosexual woman)
Legal processes	Q26 License was easy to change, but my passport was more challenging. (23 years old, heterosexual woman)
	Q27 There are no resources for legal issues about name change, so you have to independently do your research. (21 years old, pansexual woman)
	Q28 Depending on where you are born [state], the birth certificate is hard to update. (24 years old, lesbian/nonbinary)
	Q29 I have the forms, but not enough time and resources [money] to do it. (22 years old, lesbian woman)
Oregon Health Plan (Medicaid)	Q30 My trans uncle was able to get me started with insurance. He told me what calls to make to get my insurance; call OHP and talk to customer service, get income verification and documentation. OHP is perfect because it covers all hormones, except injectables; but injections are worth paying for and have less risk to organ damage. I have not gotten those covered. (26 years old, lesbian woman)
	Q31 Insurance has been very easy for me – Oregon has great Medicaid benefits for trans women. These include gender confirmation surgery, HRT, breast implants, etc. – it is through the Oregon Health and Science University in Portland – all of my partners told me about this. We also have Whitebird here in Eugene – they are very good about helping trans women transition. I was able to get HRT there the same day as my first appointment. (27 years old, Pansexual Trans Femme)
Insurance coverage (non OHP)	Q32 Insurance co-payments have been a problem. Oregon law states that is must be covered (trans care) if deemed medically necessary. The problem, of course, is that people have different versions of what is necessary. I had to, for example, justify my need for HRT to doctor who said I would not die without it. My response was that I would also not die without my eyeglasses, but I would have a horrible time getting around and being successful in everyday life. I still may need to fight things with the Oregon Health Commission. My church has also helped me with this. When I first went on HRT, it was an awakening for me, it was like, “oh, this is what feeling happy is like!” (28 years old, gender-fluid/transgender lesbian woman)
	Q33 EBMS insurance is shit to navigate; and it does not cover trans-related care (21 years old, pansexual woman)
Queer migration facilitates access	Q34 I moved here from Wyoming. While Oregon has some problems, it's incredibly good compared to Wyoming. The health care system here is so much more open [for trans women] and available; I've faced less discrimination. I choose Oregon because it's really solid for transgender people to live, especially with informed consent clinics for hormones where you don't need a note from your psychologist. I'm on my mom's insurance (from Wyoming), which doesn't cover anything trans-related; and I don't have OHP yet because I'm still not considered a resident. (19 years old, bisexual woman)
	Q35 I heard, out of the 50 states Oregon is the best place to transition. (26 years old, lesbian woman)

OHP, Oregon Health Plan; HRT, hormone replacement therapy; EBMS, employee benefit management services; WPATH, World Professional Association for Transgender Health.

Furthermore, participants believed that in Oregon discrimination existed in “coded” hidden ways—especially in school, housing, and employment. As a 19-year-old bisexual woman noted:

In Oregon, I've experienced more coded discrimination… where a person doesn't necessarily know that what they are doing is discrimination, but to them I seem foreign, scary and different. A lot of people don't treat me as a true woman because I don't pass as well.

Coded discrimination consisted of in-direct, “in-between the lines,” microaggressions that were more difficult to confront than blatant transphobia. As a 29-year-old bisexual woman poignantly stated, “There are few social options for me in Eugene, OR – it is not the queer mecca that people think.” Table 2 includes additional narrative excerpts representative of this theme (Q6–Q13). Most participants reported experiencing employment discrimination, ranging from being “dead-named and sired” to being fired. In both employment and housing situations, several transgender women reported being labeled as sex workers and “troublemakers” because of their gender (Q6–Q8). For some participants, these experiences with discrimination and violence resulted in mistrust for law enforcement (Q14, Q15). Therefore, although participants perceived Oregon to be relatively “trans-friendly” and attractive because of its policies, they experienced “death by a thousand cuts,” “coded,” and direct discrimination.

### Access to gender-affirming health care

Participants explained that the process of applying for a legal name change was perceived as “easy.” It involved an application, an application fee, and posting the intent to change their name for 2 weeks in the basement of the courthouse, so that anyone who wanted to dispute the change had a chance to do so. Most participants (60%) reported completing the legal name change process (Q26–Q29).

Additionally, participants described multiple barriers and facilitators related to use of OHP for receipt of gender-affirming services. A 26-year-old lesbian woman weighed her experience with OHP:

My trans uncle was able to get me started with insurance. He told me what calls to make to get my insurance; call OHP and talk to customer service, get income verification and documentation. OHP is perfect because it covers all hormones, except injectables; but injections are worth paying for and have less risk to organ damage. I have not gotten those covered.

Here it is also important to highlight the role of “family of choice” (in this case her trans uncle) in helping this participant navigate her health insurance. Participants who were able to access hormone replacement therapy (HRT) were able to do so through informed consent, which required fewer visits to specialists to verify that they were experiencing gender dysphoria.

Some participants who were not able to access informed consent hormone therapy expressed frustration in their process of trying to figure out the health system. As a 21-year-old bisexual woman explained,

I had to figure out the steps. First, I found a good gender therapist, but I had to pay out of pocket because it wasn't covered in my town. I had 5 sessions but the therapist couldn't diagnose me with gender dysphoria, so I had to see a psychiatrist in Eugene to get a letter with the diagnosis. Then I had to see an endocrinologist in a women's health care clinic and a primary care physician who misnamed me.

One of the most important challenges reported in this study was access to specialist physicians (Q16–Q20), which were often located in urban centers that were difficult to access for transgender women living in smaller towns. Multiple barriers were reported relative to gender-affirming health care services, with insurance-related issues being prominent. Despite Oregon's Medicaid expansion, 20% of the sample had insurance that did not cover hormone therapy. One participant provided a succinct summary of the problem.

Insurance co-payments have been a problem. Oregon law states that it must be covered (trans care) if deemed medically necessary. The problem, of course, is that people have different versions of what is necessary. I had to, for example, justify my need for HRT to doctor who said I would not die without it. My response was that I would also not die without my eyeglasses, but I would have a horrible time getting around and being successful in everyday life. I still may need to fight things with the Oregon Health Commission. My church has also helped me with this. When I first went on HRT, it was an awakening for me, it was like, “oh, this is what feeling happy is like!” (28 years old, gender-fluid/transgender lesbian woman)

Ten percent of our sample had undergone “bottom surgery” (i.e., vaginoplasty). Some of their challenges in this regard included: having “to travel 1 hour and 45 minutes by car or 3 hours by public transit” (22 years old, pansexual trans woman), “for surgery it is pretty difficult. It takes several years on the waitlist, and it's like only one doctor takes OHP” (24 years old, lesbian/nonbinary), and “having to educate the doctors about transgender women's health care and transition care” (32 years old, lesbian woman). Access to electrolysis was a salient medical challenge that limited access to bottom surgery, as noted by several women in our sample (Table 3, Q24, Q25).

## Discussion

This study describes the SDH that shape access to gender-affirming health services in Oregon. All of the transgender women in our study had access to health care, although access to gender-affirming health services were limited by type of health insurance (e.g., being on their parents' insurance in another state as opposed to the OHP/Medicaid), coming from a nonurban location (outside of Portland and Eugene) without the medical infrastructure specializing in gender-affirming health care, and being disconnected from networks of transgender communities with knowledge of how to navigate the health care system. Currently, no studies document the social experience of transgender women seeking health services in Oregon, and few studies have documented trans people's experiences accessing other type of services.^40,41^ Therefore, this study provides an important qualification of access to gender-affirming health services in Oregon with the limitations imposed by social discrimination and lack of information to navigate available services.

The SDH that emerged as critical to implementing and utilizing gender-affirming health services included gender equity in access to employment, housing, security/safety, and in social support systems. Many participants experienced “coded” discrimination (e.g., meaning indirect or between the lines or “death by a thousand cuts”), as well as more direct discrimination, which limited their work and educational opportunities and their overall social resilience. In employment contexts, our participants experienced microaggressions (e.g., transphobic jokes, being asked if they had bottom surgery, and being labeled as the “troublemaker”), direct discrimination (e.g., being fired), and harassment (e.g., sexual and police). Direct and indirect discrimination also threatened their housing status, another major SDH. Discrimination sometimes came from unexpected sources (e.g., “gender equity floor” of college dorm, other trans people, and reproduced by transgender women themselves to “pass” and not be singled out). Similarly, other research has found that limitations to employment, education, housing, and security make transgender women vulnerable to negative mental health outcomes, human immunodeficiency virus infection, and substance use and has limited their access to health services.^1,26,28,42–44^ Our study advances this literature by indicating that even in the context of affirming state-level policies, social discrimination continues to deny the realistic access to gender-affirming care.

It is important to note that nearly one-third of our sample had moved to Oregon from neighboring states because they were seeking a more welcoming social environment and greater access to gender-affirming health care services. This may reflect the phenomenon of queer migration or the movement of LGBTQ+ people from origins where they perceived more oppression to destinations that pulled them because of the welcoming social and legal environment.^45^ Leaving home can result from being expelled from or rejected by biological families, and queer migrations often lead to the establishment of “families of choice” in the destination.^45–47^ In addition to moving to Oregon from neighboring states, several participants had lived in rural areas of Oregon (e.g., Eastern and Southern Oregon) and moved to Portland and Eugene to access gender-affirming health services from specialists. Establishing new social networks can offer trans women positive social support, role models, and coping skills, while at the same time surviving in a new place, finding work, and securing housing can lead to vulnerability-inducing survival strategies, such as sex work and coping through drugs and alcohol.^48,49^

The study findings have several limitations. Through convenience sampling, most participants recruited identified as White. Although this reflects Oregon's racial composition, our difficulties in recruiting transgender women of color is likely due to their higher levels of marginalization. Our recruitment strategy included posting flyers at venues like clinics, bars, and other places that key informants mentioned as hangouts with trans clientele; it also included snowball recruitment in which participants posted the flyer on social media. Future studies in Oregon that focus specifically on people of color are necessary to explore the intersection of racism, xenophobia, and transphobia.

These qualitative findings suggest a few directions for more in-depth investigation of structural-level factors that influence the health of transgender women. For example, the findings suggest that even in this scenario there is great need for assistance programs to navigate and advocate the use of available health services. Medical-legal partnerships (MLPs) may reduce strain of navigating systems that govern access to health services.^50^ MLPs have been useful in improving linkage to prevention, treatment, and care for legally disenfranchised groups.^50^ Due to the legal issues inherent to navigating insurance benefits for transgender women, research should examine the benefits of coupling medical and legal services. As the first study to document the experiences of transgender women seeking gender affirming health services in Oregon, our research opens the way for improvements in services that are more realistically accessible in this context. In states like Oregon, where the population is concentrated in a few metropolitan areas, outreach to transgender populations potentially living in isolated rural areas of the state may be improved through remote access to tele-medical and tele-paralegal service.^51^ Future research may include comparisons with other states, such as Nevada and Colorado, which (1) are rated as having a policy environment that is highly gender affirming, (2) have neighboring states with discriminatory and nonaffirming policy contexts, and (3) have population density patterns similar to Oregon's.^29,52,53^

## Abbreviations Used

HRT: hormone replacement therapy
MLP: medical-legal partnership
OHP: Oregon Health Plan
SD: standard deviation
SDH: social determinants of health
